# A feminist neomaterialist exploration of bariatric surgery in Canada

**DOI:** 10.1177/1357034X261480061

**Published:** 2026-09-11

**Authors:** Meredith Bessey, Carla Rice

**Affiliations:** 1 Department of Family Relations and Applied Nutrition and Re•Vision: The Centre for Art and Social Justice, University of Guelph, ON, Canada

**Keywords:** bariatric surgery, interviews, new materialism, misfitting, debility, fat studies, affect

## Abstract

Rates of bariatric (weight loss) surgery have risen sharply in recent years as interventions into fatness have become increasingly medicalized and debates surrounding it binarized. We adopted a feminist neomaterialist perspective to challenge binary framings of weight loss surgery experiences still dominating the field’s conventional and critical approaches by thinking relationally about entanglements of discourse and materiality. Within our neomaterialist framework, we used conceptual tools from misfitting, debility, and affect theory to analyze interviews with 21 women residing in Canada who had surgery at least 1 year prior. Using reflexive thematic analysis and thinking with theory, we generated three processually oriented themes: the force of pre-surgical (mis)fitting, (un)certainty of a (non)fat future, and post-surgery as a liminal space. We argue that while surgery is often presented as a pathway to normality and “fitting in,” ambiguity and liminality emerge as hallmarks of post-surgery experience, unsettling binaristic portrayals of these surgeries.

## Introduction

Bariatric surgery (colloquially, weight loss surgery) has grown substantially in frequency, with surgeries in Canada increasing by 330% between 2009 and 2018 (Obesity Canada, 2019). Typically, patients^1^ must have a body mass index (BMI) above 40 without comorbidities, or above 35 with certain comorbidities (e.g. type II diabetes), to qualify for surgery within most provincial healthcare systems, although eligibility criteria vary provincially (e.g. Nova Scotia Health, 2024; Ontario Bariatric Network, 2024). Canada’s under-resourced public system is often slow to provide access, leading to long wait times in some provinces (Obesity Canada, 2019); private clinics performing the procedures, both domestic and international, offer reduced wait times but high financial costs (Martin et al., 2011). Sleeve gastrectomy and gastric bypass (or Roux-en-Y) represent the most common publicly available procedures, with fewer provinces offering gastric banding (Obesity Canada, 2019). While these surgeries broadly have the same goal—restricting intake and modifying metabolic processes to induce weight loss and impact health—they attempt to achieve this through different means. Considered to work primarily through restricting intake, sleeve gastrectomy involves removing a large portion of the stomach, leaving behind a narrow tube; gastric band similarly restricts food intake via a surgically implanted restrictive device that is inflated/deflated to adjust stomach size (Piche et al., 2015). Roux-en-Y entails creating a small pouch from the stomach and connecting it to the small intestine, bypassing part of the digestive system; it is considered a hybrid restrictive/malabsorptive surgery (Piche et al., 2015). In recent years, glucagon-like peptide-1 (GLP-1) receptor agonists, such as Ozempic, have become more commonplace interventions for “obesity,”^2^ reducing weight by delaying gastric emptying time and diminishing hunger (Popoviciu et al., 2023).

Despite the introduction of GLP-1 agonists, medical logics (as of now) still consider bariatric surgery the “gold standard” for obesity treatment (Covey, 2025; MacLellan and Johnson, 2021). In contrast, fat studies scholars have long located the impulse to “cure” fatness in the cultural pathology known as fatmisia—powerfully negative constellations of cognitions and affects (hate, disgust, fascination; Rinaldi et al., 2020) that put fat health and life at risk (Brown and Herndon, 2020). Obesity science and fat studies still tend to frame reasons for, and outcomes of, surgery in binaristic terms—as a problem of physical bodies *or* of sociocultural worlds. Gard (2011), for example, draws scholarly attention to the two poles, the “alarmists” (obesity scientists, related others) and the “skeptics” (fat studies, critical weight scholars), while emphasizing heterogeneity within each in an effort “to move beyond the idea of there being two camps in debates about obesity” (p. 38). Although his articulation critiques dualism, it stops short of offering a less binarizing alternative, and dualistic thinking persists. Some obesity scientists have shifted away from conceptualizing obesity as an individual failing toward understanding it as a disease with multifactorial causes (Wharton et al., 2020), although this framing remains contentious (Steele and Finucane, 2023). Moreover, while the field increasingly *acknowledges* weight stigma’s contribution to adverse psychosocial and physiological outcomes often attributed to higher weight (Puhl and Suh, 2015), most large-scale studies still fail to *investigate* how such stigma intra-acts^3^ with other sociopolitical forces—neoliberalism, structural racism, and sexism—in producing those health outcomes (Sun et al., 2022). As a result, poor health remains tethered to body size itself instead of to the cumulative physiological and psychological burdens of living as fat in a fatphobic world.

At the other pole, the skeptics, including fat studies and critical weight scholars, share a critical, fat-affirming approach to understanding fatness and health, although critical weight researchers often engage more comprehensively with scientific evidence underpinning the obesity-as-disease paradigm (Bombak, 2014). In taking a politicized view, fat studies scholars mainly focus “on the discursive construction of fat bodies rather than the materiality or agency of bodily matter” in an effort “to distance from essentialist discourses” (Warin, 2015: 48), and have tended to adopt a firm anti-surgery stance that often excludes surgery recipient voices (Brown and Herndon, 2020). Yet there are signs of movement in critical scholarship: Throsby (2012a) positions her weight loss surgery research squarely within a critical framework, arguing that the decision to undergo surgery cannot be understood simply as capitulation to anti-fatness, but “as a complex interaction of interests, desire and power relations,” inseparable from, yet not reducible to, anti-fat ideologies (p. 107). Recently, scholars have advanced more entangled approaches, highlighting the misfit many recipients encounter between their pre-surgery body and the world as a motivating factor for surgery (Meleo-Erwin, 2015), and the reduced stigmatization some describe due to appearing or feeling healthier, despite acquiring new symptoms post-surgery (Stevens, 2020). Following Throsby (2012a), we view “taking patient demand for, and endorsement of, obesity surgery seriously” not as a departure from critical fat politics, but as integral to it (p. 107). Building on these approaches, we seek to open analytical space for dimensions of surgical experience that exceed the terms of the alarmist-skeptic divide, moving toward more relational, less binarizing accounts.

Drawing on interviews with 21 surgery recipients, we use a neomaterialist framework to surface and suspend binaries that organize understandings of surgery experiences. Employed by a small but growing number of critical fat scholars (e.g. Brewis et al., 2017; Whitesel and Shuman, 2016), neomaterialist approaches orient to fat’s materialization as relational, processual, and emergent, rather than pathological, as metamorphosing through ongoing relations with a lively socio-material world (Slatman, 2021). Feminist neomaterialism inherently refuses entrenched dualisms (Blackman, 2016), adopting a monist ontology that troubles divisions between scientific and critical claims to show complex entanglements of discourse and matter. Our research extends these conversations by examining the politics of a rapidly changing medical landscape and its implications for fat people, where bariatric surgery and, more recently, pharmaceutical interventions have become increasingly common (Popoviciu et al., 2023).

Given fat studies’ long-standing commitment to shifting epistemic authority to fat people, loosening the field’s boundaries invites a more nuanced consideration of how (some) weight loss technologies might benefit (some) fat lives. Simultaneously, this orientation challenges obesity researchers to engage differently with questions of health and impairment by considering how weight stigma, adverse socioeconomic conditions, medical interventions, and other sociomaterial forces materially or physiologically impact bodily capacities and contribute to forms of debility frequently attributed to fatness itself. Together, these moves open space for both fields to more meaningfully engage with the intra-activity between the social and material that co-constitute bodies, surgeries, capacities, and constraints, potentially benefiting fat lives. We see our neomaterialist approach as carving a pathway through the impasses between culturalist and biological determinist positions still haunting these fields, where experts replay well-worn dialectical debates, ignoring or obscuring how biology inevitably comprises “a potentiality, which is never pre-social or pre-objective” (Blackman, 2007: 17).

## Entanglements of theory and method

To generate a complex picture of participants’ surgery trajectories, our theoretical framework develops the concepts of misfitting and debility in the context of debates within the fields of affect study and new materialism. People who inhabit bodies typically coded as “obese” often confront “a social and built environment mismatch” (Meleo-Erwin, 2015: 2), reflective of the neomaterialist concept of (mis)fitting, or “encounter[s] in which two things come together in either harmony or disjunction” (Garland-Thomson, 2011: 593). While some research has analyzed how misfitting constructs bariatric surgery as a viable response, researchers have rarely considered the ways misfitting persists and moves post-surgery (Brewis et al., 2017). From embodied misfitting experiences, we expand outwardly to query how embedded or structural forces such as neoliberal capitalism and anti-fat rhetoric also operate as surgery catalysts, in part, by producing/exacerbating health problems associated with fatness (Puhl and Suh, 2015). We conceptualize misfitting as produced through physical environments alongside social norms, everyday encounters, and affective atmospheres that materialize misfits between bodies and worlds, which generate ontologies of othering and attendant desires for fitting. Here we also draw on the concept of debility to theorize the effects of socio-material forces on bodies they target. Described as ongoing destructive forces that wear bodies down and subject them to “slow death” (Berlant, 2007: 754), debility in our application refers to anti-fat violences circulating in every sphere of daily life (housing, healthcare, family relations) that materialize fat as non-normative and pathological (Bahra, 2023; Puar, 2017). Thus, debility is not inherent but is created through structural forces that cannot be disentangled from interpersonal or internalized fatphobia. Folding debility into our analysis enables us to carefully consider the grave material, affective, and social impacts of misfitting for fat bodies—to understand why misfitting *matters.*

Misfitting and debility alone, however, do not quite capture the degree of harm that many fat people endure. Affect is embodied, relational, and material, and sticks to bodies, turning fatmisia from something ephemeral into a felt reality where the “aim . . . is to expunge fat life” (Ahmed, 2014; Kotow, 2024: 49). Thus, layering affect theory allows us to more clearly track the operations of fatmisia on bodies, including how biomedical discourses about obesity operate as powerful affective forces, intensifying concerns about fatness and health (Gailey, 2022). In this context, choosing to undergo bariatric surgery becomes sensible—affectively assuaging—and a way of bringing the body into control, rather than being weighted with (im)morality. Dominant discourses construct fat bodies as disorderly, dysfunctional, and/or unpredictable (Ekman, 2023); in response, medical systems construct surgery as a way of putting bodies into order and of imposing predictability. However, bodies and surgical interventions are inherently unstable and unpredictable, calling into question this construction. For example, predictors of surgical success remain unclear, as do mechanisms underlying certain risks, such as the development of alcohol use disorders (Belligoli et al., 2020; Geliebter, 2025); as such, what intervention is considered “best” is also in flux.

These theoretical concepts were inseparable from our methodological approach and researcher positionalities. This study formed part of Meredith’s doctoral dissertation, supervised by Carla. I, Meredith, am a 36-year-old, white, queer, neurodivergent cis woman trained as a dietitian who now works as an assistant professor in nutrition and dietetics. I experience thin privilege and have lived experience of body- and eating-related distress. I, Carla, carry professional and political experiences as a former therapist and fat activist as well as embodied knowledge gained through moving through the world for more than six decades as a fat, not-fat, and fat again, mad, queer femme. These shifting embodiments attuned us to the profound force of fat hatred and people’s desires to escape it, informing how we listened to, felt, and interpreted participants’ surgical narratives.

Fat studies has at times taken a doctrinaire position in which engagement with weight loss technologies is read as a betrayal of fat liberation and life (Brown and Herndon, 2020). Our analytic aim was not to adjudicate this (understandable) stance, in part since we ourselves have taken this position, but to create space to think-feel with the messy, unresolved, often shame-laden dimensions of embodied fat experience and body modification. Working with thinking with theory (Jackson and Mazzei, 2012) and reflexive thematic analysis (Braun and Clarke, 2022), we experienced the data collection and analytic processes as affectively charged. At times, we each felt sadness and frustration in response to participants’ accounts, but also discomfort, disappointment, and even existential fear and dread for fat existence, when their stories did not conform to unequivocally negative narratives of surgery. Attending to these reactions revealed how easily good/bad binaries are reproduced, even in work committed to troubling them.

Through ongoing discussion and deeper engagement with feminist neomaterialism, we learned to sit with these deeply discomforting affects and consider more carefully the entangled, power-infused terrain in which surgical decisions take place. One fraught interview proved particularly impactful, where a participant’s expression of significant internalized fatphobia produced agitation and embodied sensations of heat in Meredith. In a subsequent conversation, Carla posed a question that redirected our analysis: were biomedical interventions always a site of harm, or might they sometimes constitute a liveable response in the face of pervasive fat hatred? This moment pushed us to dig deeper, sharpening our recognition that both surgery and people’s desires for it emerge through entrenched fatmisia within a field of profoundly unequal power where biomedical logics dominate fat-affirming counterpoints. Suspending binaries required resisting moral certainty and learning to open space for all types of surgical stories alongside upholding our commitments to fat justice. Guided by these theoretical and methodological orientations and our experiential grapplings, we now trace how participants’ surgery experiences take shape through entanglements of misfitting, debility, and affect across pre‑ and post‑surgical trajectories.

## Themes

We interviewed 21 women who had had surgery, most of whom were white except for two participants who were Métis and Middle Eastern, respectively. Participants ranged in age from 26 to 61 and had received surgery between 1 and 15 years ago. Seven participants had a sleeve gastrectomy (one later had it revised to a Roux-en-Y), 11 had a Roux-en-Y (one had since had it reversed), two had a mini gastric bypass, and one had a gastric band that was later revised to a Roux-en-Y. Participants used several terms to describe their current body size, including curvy, average, plus-size, mid-size, straight-size, and fat.

Our analytic process generated three themes: the force of pre-surgical (mis)fitting, (un)certainty of a (non)fat future, and post-surgery as a liminal space. These themes capture embodied processes of people’s surgical journeys, with the first theme focusing on pre-surgical misfitting as powerful motivation for surgery, the second drawing attention to uncertainties that mark interlocutors’ surgical decision-making processes, and the third bringing us into intimate contact with participants’ post-surgical embodiments, foregrounding instabilities and liminalities that persist even following the intervention.

### The force of pre-surgical (mis)fitting

Our first theme captures participants’ experiences of misfitting before surgery, including how a misfit with material aspects of environments pre-surgery often functioned as a motivating factor for undergoing surgery. For example, Elizabeth noted that she “needed to fit into the MRI [Magnetic Resonance Imaging] machine [to assess chronic back pain], and I needed that to happen as quickly as possible, ‘cause I was in pain constantly.” Elizabeth’s pain was properly assessed following surgery and subsequent weight loss, but “fitting” also came with resentment at the carelessness with which medical professionals treated her pre-surgical body, an experience she was still processing 3 years post-surgery. Nancy’s similar misfitting experience prompted frustration: “maybe you guys should develop technology that works on people that are overweight instead of blaming the weight problem . . .” The medical complex constructed Elizabeth’s and Nancy’s bodies as a barrier to healthcare rather than identifying the misfit between their bodies and the built environment as the issue requiring resolution.

Echoing previous research (e.g. Brewis et al., 2017), participants also encountered misfitting outside healthcare, in recreational spaces, on airplanes, and even at rest, all of which carried an intense affective load. For example, Amy highlighted the affective weight of doing “simple things” like sourcing furniture that she could feel confident would support her in meeting one of life’s necessities: sleep.

. . . I sleep on a mattress on the ground still . . . because I was scared to make my bed at my heaviest. So, my bed’s still sitting in a box in my room . . . little things like that, I was scared that I was gonna break the slats—

The Internet is rife with concerned comments from thin people about their fat friends or guests breaking furniture; Amy understandably internalized this anxiety, even if it meant making herself more uncomfortable. These everyday harms that render fat people as/at the outer boundaries of humanness adversely affect people’s wellbeing via the gnawing stresses of misfitting and existential threat of fatphobia (Puhl and Suh, 2015). Donna shared an experience of misfitting that further highlights material-affective-embodied entanglements:

[A]lways being the fat girl was never fun . . . I was out with family for dinner, and we were in a restaurant where you had to . . . shar[e] tables and booths . . . I was having a hot flash . . . and I thought, I gotta get up and get some air . . . I couldn’t get out comfortably, and this man that was sitting there, I got up and I sort of bumped him with my rear end . . . I just was a bit horrified, thinking, Oh my God, that poor man, you know, he had to stare at my backside . . . I felt really horrified in that moment, and just thought to myself . . . Well, if you weren’t so bloody fat . . .

Everyone “got a laugh out of it” and she laughed too, though it did not feel funny; Brewis et al. (2017) described the need to become the “jolly fatty” when misfitting is unavoidable. The self-blame evident in Donna’s experience might function to invisibilize violent ontologies operating on fat people, further reinforcing misfitting as a problem to “solve” individually rather than systemically/materially.

Facing lifelong weight stigmatization led some participants to become the “easy patient” (Kelsey) to avoid risking losing access to healthcare:

I just accepted [having everything blamed on weight] . . . because I didn’t wanna be labeled the difficult fat girl with my doctor . . . and I just didn’t wanna put myself in a position where I wouldn’t be able to go see my doctor . . . so, I was always compliant, and always did what I had to do. (Elizabeth)

We connect Elizabeth’s experience to pressures to meet white middle-class gendered expectations about being a “good girl,” especially as a woman whose fat already violated codes of femininity (Taylor and Hoskin, 2023). Being compliant allowed Elizabeth to partially mitigate size-based misfitting by performing class- and race-based gender norms. In addition to violating (white) feminine ideals, the fat body also maximally deviates from the species standard, enjoining subjects like Elizabeth to make their bodily selves more recognizably human through “moral discipline and self-management” (Apostolidou and Sturm, 2016: 150). Indeed, much weight stigmatizing rhetoric stems from ideas that fat people are lesser than, lazy, non-compliant, and so on (Puhl and Heuer, 2009), making those coded as fat into othered lifeforms not seen as having a place in the *human* medical system.

Further expanding on white gendered norms, Deborah Y. described herself as a “Glamazon” pre-surgery, as someone who stood out “in a room full of fat girls.” She asserted that in her experience, many fat women have an exaggerated relationship with femininity, where “we tend to be drag queens almost, in how we interact with the world,” by having great hair and makeup, “dressing well,” and behaving flirtatiously. In this gendered expression, Deborah potentially enacted “compensatory femininity” (Lapping, 2024) to redeem herself for fat’s failure to abide within white femininity’s bounds. While surgery promised a more properly feminine body, it also betrayed Deborah by creating a body that continues to misfit with femininity’s normative modes (Taylor and Hoskin, 2023). After surgery, she “was no longer the big fish in a small pond of confident, large women”; adjusting to being “just one of the crowd” propelled Deborah to seek psychological support through the bariatric clinic.

In her affect-laden encounter with a dietitian during her formative years, Allison recounts how the provider constructed her supposed inability to control her weight as a moral failure that justified her disposability, highlighting how fatmisia circulates widely as a life-destroying force:

I remember going to a dietitian . . . 10 or younger, and her just saying, Well, you’re kind of a lost cause . . . . and I remember just thinking, well, I guess this is just how I’m meant to be, right? I don’t know if that’s the actual words that she had said; I was young, but I remember just feeling, well, this dietitian doesn’t even think that I can lose weight, so, if she can’t help me, then maybe I’m just meant to be this way . . .

Allison evokes the feeling of being trapped in the ostensibly immutable space of fatness, where she, like Elizabeth, transgressed permissible boundaries of the human; surgery later came to feel like the only available solution to undo her implanted sense of excess. Emma also endured ego- and body-damaging affective encounters with a therapist who she described as being a “pretty awful person that had no patience for obesity”:

. . . she just kept telling me she didn’t see why I couldn’t get up at 5:30 in the morning and walk for 90 minutes every day and lose the weight, and then I’d be happy . . . it was horrific . . . I left there in tears every time thinking, this woman hates me and I’m a loser [laughter], and so I quit . . .

Imbuing Emma with a sense of hopelessness and of being left behind, the therapist’s unceasing anti-fat refrain possibly targeted Emma due to her perceived failure to meet hegemonic standards of a healthy self-regulating feminine subject under neoliberalism. Her therapist also seemingly clung to the promise of happiness (Ahmed, 2010) culturally awarded to women who successfully diet, the “good fatty” who does all they can to change their weight and are thus more willingly humanized by society (Gibson, 2022). Bariatric surgery ultimately offered an alternative pathway to happiness—to being perceived as acceptably gendered and human—that Emma did later take up.

Several participants reported other experiences of misfitting, including Dianne, who described being placed on “extreme” diets from age eight because she “couldn’t dress like the other kids,” and how misfitting and constant bullying at school led her to suicidality and to quitting school at 15. Her drive to fit into “social norms,” due to the intense alienation she survived, fueled her surgery desires, which, in her own words, “disabled” her. Within her family, Emma navigated anxiety-ridden relations pre-surgery:

I had a really tumultuous relationship with my parents in that they were constantly trying to . . . stage an intervention about me and my weight . . . huge, huge, huge fear of interactions with people . . . I didn’t want to be left alone with any family members.

Her family’s wish to “stage an intervention” into mundane and intimate aspects of embodied existence cut to her core by generating a deeply affective sense of not belonging within what we culturally code as the most nurturing of relations: family. Participants materialized as neoliberal misfits pre-surgery, where environments that mismatched with their bodily selves made them feel inadequate (Skiveren, 2022). Although not an easy “solution,” seeking surgery to at least partially remedy this misfit emerged as sensible—affect-relieving and practical—under these conditions.

Significantly, Elizabeth described “living as an obese person . . . [in] the society that we live in” as “one of the largest traumas in my life,” gesturing toward the fatmisia that circulates via everyday structures and relations (Ahmed, 2014), making it impossible for participants to escape the hostility, cruelty, and debility it produces. We turn now to unpack how systems and discourses construct surgery as a way to recuperate the debilitated, misfit body and its (un)certain fat future; as with cosmetic surgery, medical systems construct bariatric surgery as “the most rational solution” to the problematized state of fatness (Negrin, 2002: 25), keeping the focus on the individual rather than on the many societal, material, and affective forces that debilitate fat bodies.

### (Un)certainty of a (non)fat future

The second theme captures participant decision-making processes amid discursive (un)certainties about a (non)fat future, where adopting the dominant narrative that fat bodies cannot anticipate a good or healthy future left participants wondering whether surgery would produce an appropriately “healthy” thin one. Even if they enjoyed it now, “good health,” Chloe opined, would not last going forward, as time and living would render fat pathological despite the current absence of disease, and participants who already managed weight-related health concerns expressed worries about possible worsening health. The weight loss industry scripts and anticipates the material futures of fat people before they even engage with the bariatric system (Adams et al., 2009); participants’ stories both undo and buttress that script in various ways. Several participants gestured to approaching the surgery as preventive, with Deborah Y. stating “I wasn’t disabled by my weight, but I didn’t want to become disabled by my weight,” an outcome that she felt “comes with age.” This reflects the dominant narrative trajectory that fat leads to illness, disability, and a life not worth living (White, 2012), where fat people are frequently “haunted by the disability to come” (Puar, 2017: 12).

Many participants also feared that staying fat risked an early death. As Shaun stated:

You do not see 80-year-old obese people, and there’s a reason . . . I would like to live to be 90, and how do I do that effectively, knowing that if you’re 100 to 200 pounds overweight, you’re just setting yourself up for a lot of issues and an early death.

While she enjoyed being “super healthy . . . except for the weight” before surgery, Shaun learned to see living a long life as only possible via surgery. Biomedical discourses about obesity and health entangle with and produce affects such as anxiety and fear; in Canada specifically, these discourses are also racialized, with scientists and policymakers mobilizing the “thrifty gene” hypothesis (Poudrier, 2016) to paint Indigenous peoples as at particular risk for diabetes and obesity. Although no one in our study, including the one Indigenous participant, alluded to the thrifty gene hypothesis, the settler-colonial move to reduce the complex sociomaterial risks confronted by Indigenous peoples under settler colonialism to genetics comprises an urgent problematic for future exploration.

Despite common misconceptions about fatness always reducing lifespan, research suggests a more complicated reality where, while high BMIs typical of someone pre-surgery are positively related to higher mortality, people with BMIs between 25 and 35 have the same or *lower* mortality than those in other weight categories (Flegal et al., 2013). In addition, as noted above, most research investigating associations between BMI and mortality does not consider the health-limiting effects of fatmisia, colonialism, and other debilitating structural forces (Puhl and Suh, 2015). Taken together, the science supports participants’ health concerns in some ways, but also complicates the story. Biomedical discourses remain deeply colonial, defining health reductively and privileging some, often physical, aspects over others (Kendall et al., 2019). As Indigenous scholars have noted, a holistic, decolonized approach, including to health, aligns with a new materialist frame (e.g. De Line, 2016) insofar as both approaches orient to wellbeing as processual and co-shaped through symbolic, structural, and organic forces.

In addition to Dianne’s experience, discussed above, another participant spoke about experiencing suicidality related to the unliveability of the world as fat, gesturing toward the violence of fatmisia. Dina noted that

by the time I was 13, 14, 15, I . . . made a deal with myself that if I did not find a solution, I was going to kill myself at some point; it was non-negotiable. It was interesting because I didn’t perceive myself to be suicidal; I was not depressed at all, I was actually a pretty happy kid, but I just understood that . . . a fat life for me was not a life worth living, full stop.

Participants like Dina struggled in a world that did not see them as belonging to the human race, internalizing feelings of existential non-belonging. At age 19, Dina underwent bariatric surgery, which she described as a “metamorphosis” and a “renaissance of self,” suggesting that bariatric surgery serves “as an act of generation” (Shildrick, 2008: 36). However, Dina also noted that she “felt like a skinny person inside my whole life,” suggesting that the surgery did not so much materialize a *new* self as a previously *imaginary* one, one which allowed Dina to see herself as aligning with the human family. Although this framing could be seen as privileging the material over the affective/imaginative, we interpret it as exposing biomedicine’s privileging of materiality and imposition of the mythical norm of embodiment (of which thinness is part), where experts pay little attention to the emotional impact of surgeries as long as patients become smaller.

Concerns about future wellbeing intersected with often-long wait times described above to move five participants toward private surgical clinics. These participants paid up to C$20,000 for their surgeries (2017–2022), making reduced wait times something only people with financial privilege can access. Alex noted:

[T]he 60-month waitlist is, at that point, I will be pushing 60, and I don’t want to wait that long for surgery. In the meantime, my asthma’s terrible, my MS [multiple sclerosis] is getting more stumbling . . . I literally couldn’t walk to the end of the block without starting to wheeze . . . so, that’s when I started exploring going to Mexico for surgery . . .

In wanting to “get this over with” to “get healthy now,” Alex’s account speaks to the ways neoliberal technocapitalism regulates bodies and psyches, wherein the psychologization of control turns struggles with power into struggles with the self, into battling one’s embodied being and future to make life liveable in the here and now (Shildrick, 2015). Sociomaterial realities surrounding disability also inflect Alex’s account: while she had resisted when her doctor suggested bariatric surgery 5 years earlier, the worsening of her MS, and the inadequate health and social care she received living at the nexus of two devalued differences, weight and disability, prompted her pursuit of surgery.

For Hailey, surgery has similarly given her confidence in a future where things get better:

there was one moment that really struck me . . . a couple weeks post-surgery . . . I kind of realized that, Oh, it’s not all downhill from here . . . now I feel like my health and my strength and my stamina can get better . . .

Before surgery, Suzanne assumed that she “was gonna be healthy and skinny . . . and finally be happy,” and Laura was optimistic that surgery would offset risks related to her weight. For these women specifically, and participants generally, surgery promised an improved life. Unfortunately, for Suzanne, her surgical outcome was “not as great as I thought it would be,” and Laura felt like she’d “kind of ruined it” by regaining most of the weight, largely due to alcohol dependency she developed post-surgery, a not uncommon outcome of bariatric surgery (Esin et al., 2023). Suzanne and Laura have not experienced complete fulfillment of the promise of happiness and health that surgery offered them. However, for other participants, these promises *were* fulfilled, with Chloe stating, “I think it’s pretty much . . . the best version of what I had expected.” This fulfillment still often carried elements of ambiguity or liminality, however, as we explore below.

Finally, most participants acknowledged and accepted a certain amount of surgical risk to offset emotional and physical risks of continuing to be fat; Laura approached surgery’s risk/benefit trade-off practically, by re-doing her will and talking with her adult children, but did not feel especially anxious or nervous. Lisa S. felt similarly:

I knew about . . . different possible negative things that could go [on] . . . but just focused on . . . the good stuff . . . I thought, well, I have to do this, and I’ll just have to deal with whatever . . . ‘cause it really just felt like it was life and death, I . . . physically felt like I was slowly dying every day and so, I thought, Yeah, I just gotta do it.

Not as concerned about the potential risks of surgery as about the perceived “life and death” risks of remaining fat, Lisa evokes Berlant’s (2007) “slow death” and defames fatness itself as the cause rather than fatmisia and other intersecting socio-material forces that produce fatness as unlivable.

By undergoing major surgery, participants gambled on a future that felt worth the risk, in part to attain a “de-stigmatized social identit[y]” (Felder et al., 2016: 413). Even for someone like Lisa M., who had already had two “failed” bariatric surgeries, hope remained that another revision would finally “work.” Despite contrary experience, the possibility of another surgical fix created a sense of cruel optimism (Berlant, 2011) that things would turn out differently this time (Kotow, 2024). Berlant (2011) theorizes “cruel optimism” as a harmful relation that forms “when something you desire is actually an obstacle to your flourishing” (p. 2), naming eating as one such desire that many people engage in to gain relief from the stress of oppressive structures, but that interferes with their path to thinness or, in Berlant’s terms, flourishing. However, in line with scholars like O’Leary (2022), we wonder whether bariatric surgery and other weight loss interventions instead produce cruel optimism by promising a solution to abjection that cannot be fully realized. “Unrealistic” expectations that surgery will fix everything, demonstrated in other bariatric surgery research (e.g. Felder et al., 2016), implicate bio-societal norms that value controlling fatness over preserving (quality of) life. The weight loss industry materializes these “unrealistic” expectations, which, just as surgery is a practical response to fatmisia and misfitting, are in fact sensible (affectively relieving; rational) responses to discursive constructions of surgery.

In sum, participants reported fears about their future as a fat person, current or future debility, and slow death, with surgery offering a sense of cruel optimism about the possibility of a better (nonfat) future. Whether this better future came to fruition is explored below.

### Post-surgery as a liminal space

The final theme conceptualizes post-surgery as liminal; a neomaterialist framework enables us to trace processes of transformation across and through fluid boundaries between categories or embodied states. Media representations of bariatric surgery (Glenn et al., 2013), which anti-obesity discourses buttress and bariatric programs fail to sufficiently challenge, frequently leave the perception that surgery will move people from the liminal state of fatness into a legible bodily form post-surgery. Fatness is thus considered an impermanent condition, a temporary stopping place on the way to someone’s *actual* thin body (Kyrola and Harjunen, 2017), as Dina describes above. However, participants’ narratives complicate this discourse by revealing ongoing liminality where post-surgery bodies occupy a uniquely heterogenous, unsettled space. While fat’s liminality is typically theorized in temporal terms, as oriented toward eventual resolution through weight loss, our conceptualization of post-surgical liminality foregrounds a different dimension of embodied experience: the persistent misalignment of post-surgical bodies with medical-social norms that presume bodily coherence, linear recovery, and definitive surgical success. Whereas fatness is culturally viewed as temporary, the post-surgical body often remains suspended in an in-between state indefinitely. Weight loss surgery, then, does not resolve liminality; it reconfigures it. The bodily transformation it makes does not always align with individual expectations, societal imaginaries, or medical-conventional standards (Jiretorn et al., 2024; Throsby, 2012b). Instead, it brings a new bodily form into the world, one that remains in process, governed through ongoing evaluation, and rendered legible through continual negotiation with normative frameworks that cannot fully account for it:

Without the yoga pants, I don’t look like you and people look at you, and they don’t know what to make of you. You’re not fat, that’s for sure, but you’re not skinny, because there’s this thing on you . . . it looks weird, it’s kind of thin and it hangs, your loose skin and the tissue (Dina).

Participants’ post-surgical bodily differences encompassed loose, overflowing skin which can be read as a sign of “former fatness,” as a sign that one’s body continues to misalign with normativity despite the intensive intervention it has undergone (Throsby, 2012b: 11).

Some participants found that additional skin removal surgery provided a better “fit,” highlighting the fluid and liminal nature of (mis)fitting; the post-bariatric surgery body could be considered an “incomplete body project” that requires further intervention to align with normativity (Katsulis, 2018: 154). As Kelsey noted:

I will say that [skin removal] was way different on my brain than almost anything else, because . . . this area of biggest insecurity was now . . . this looks really good . . . it was right after I had . . . that surgery where the whole polyamory thing kind of began because I was, Oh, now I feel confident, I feel like I’m wearing clothes that I’ve always wanted to wear and . . . as strange as that is, taking that skin off, that was such a big shift.

More so than significant weight loss, skin removal provided Kelsey with a renewed sense of freedom and confidence to open her marriage for the first time. For other people, like Shaun, skin removal surgery also meant a continual negotiation of how they fit:

. . . one of the negative repercussions of surgery is my golf swing completely changed because my body changed and so, I was really annoyed that I had to relearn my swing, and now I’m annoyed because I just had skin removal surgery and I’m gonna have to relearn my swing again this year [laughing].

Fitting emerged as an evolving process for these participants wherein the body and its relationalities continue to shift post-surgery, extending existing research (Brewis et al., 2017; Meleo-Erwin, 2015) to show ways that (mis)fitting moves post-surgery.

Significantly, the liminal space that many post-surgery clients occupy encompasses uncertainty about weight loss outcomes, as Lisa M. describes:

I really don’t think [clinics] do a good enough job of explaining . . . that you basically will gain back all of your weight in a year or two or three or four naturally . . . . you get problems on both ends of the spectrum ‘cause . . . some people get super thin and really unhealthy, and then other people gain it back and rarely do you find someone who has actually kind of hit the sweet spot where it’s, I lost the weight I needed to lose and I’m healthy and I’m happy and I’m maintaining. I haven’t met anyone like that yet.

Some participants, like Hailey or Alex, seemingly fit Lisa’s definition of “hit[ting] the sweet spot” while other narratives reflected this ongoing challenge to find a bodily balance post-surgery. In addition to loose skin, participants described hair loss, gaunt faces, and significant gastrointestinal changes, such as “dumping” syndrome (diarrhea, nausea, etc. related to rapid gastric emptying), reflux, and impacts on hunger/satiety cues (Quercia et al., 2014). Often framed as “temporary,” these effects can persist or even require revisional surgery (Groven et al., 2013). For example, Amy had severe reflux since her surgery a year prior but remained effusive in her praise for the intervention and her healthcare team. Nancy dealt with intense reflux for a year-and-a-half after her initial sleeve gastrectomy until a revision to Roux-en-Y, also dealing with other complications like hernias. However, both Amy and Nancy vastly preferred living with these outcomes to the visceral-psychic-affective injuries they endured before surgery. As Stevens (2020) notes, this trade-off is worth it for many surgery recipients, as was seemingly the case for many, although not all, participants in this research. Suzanne, for instance, saw surgery and its consequences as a high cost to pay:

I mean, my arthritis is in remission, yay! [laughter] . . . and I’m not on the CPAP [for sleep apnea] anymore, wonderful, but I went through some very bad health issues with this surgery . . . it really destroyed my mental health . . . I don’t feel like I can celebrate the way I look right now.

These experiences are reminiscent of Murray’s (2009), who writes that while bariatric surgery resulted in her having a more normative, assumedly healthy, appearance, a “‘dis-abled’ lived experience” (p. 156) became an unwelcome facet of her new post-surgical form.

Relationships with food surfaced as another source of continued liminality: participants like Hailey experienced liberation from “food noise” (“constant and persistent thoughts about foods and eating”; Hayashi et al., 2023: 2) while others found themselves still preoccupied with food choices. Deborah G. had expected that surgery would “be the magic fix” for her relationship with food, but has realized since surgery that she might never be free of food stress; this follows other research showing that negative relationships with food often persist after surgery (e.g. Groven et al., 2018). Allison suggested that the constant preoccupation she now experiences is likely “normal” for people who have always lived in a smaller body: “Do non-overweight people think that way too? Probably . . .” In some ways, this meant that she felt more recognizable as a normative human post-surgery and perhaps “fit” better with the idea of a conventional, in-control woman. As Throsby (2008) notes, bariatric surgery can be viewed as allowing people to “diet like a normal person” (p. 127); rather than being a solution to lifelong dieting, surgery can open the door for an increasingly entangled relationship with it.

Even experiences of sociomaterially fitting post-surgery did not come without complicated, ambiguous feelings, which sometimes served to highlight how the world makes the fat body misfit. As Dina pointed out:

[I]t’s very, very difficult because when you’re fat, and you’ve been fat your whole life, you can’t tell . . . it’s only after you lose the weight do you see the difference . . . there [was] discrimination in every moment of my life.

Fatmisia folds into the very structures of society (e.g. MRIs and beds) and operates as a circulating affective-discursive force that permeates almost every moment of a fat person’s life, undermining their claim to humanness through subjecting them to an existential sense of wrongness. In contrast, participants noted improved treatment in various contexts post-surgery; for example, doctors were more inclined to listen to Suzanne’s experiences rather than jumping quickly to recommend weight loss, and Kelsey felt people’s increased comfort and ease looking at and engaging with her directly, “now [that] I’m a socially acceptable size.” Several participants expressed frustration at the improved reactions they receive/d post-surgery; after a lifetime of being treated terribly because of her weight, Dianne shared how her family thought she “looked spectacular” post-surgery, finally inviting her to events. Being “the shiny new thing” faded, however, as she became further disabled and isolated by surgery’s long-term impacts. Deborah Y.’s experience also highlights the fickle nature of fitting; they dealt with significant health consequences from surgery and regained most of their lost weight but were still glad they had the surgery: “I’m glad I got the feeling of what it’s like to be thin . . . it was lovely to actually feel that way.” While she would not choose to have the surgery again, she still experienced a certain amount of wonder, pleasure, and satisfaction with temporarily fitting, even though it was ultimately fleeting and detrimental.

## Conclusion

Misfitting produces ontologies of violence for people who do not fit or who violate the norms of humanness, contributing to the debilitation of fat embodiment and life. Bariatric surgery recipients hope that surgery will enable them to move into the realm of normative humanness, but liminality remains post-surgery, where surgery puts a new, heterogenous form into the world. Typically, there is limited space offered within fat studies/activism to explore the complexity, ambivalence, and even pleasure that can accompany weight loss surgery in a fatmisic world (Brown and Herndon, 2020). Our analysis emphasizes the importance of scholarship that accounts for, and takes seriously, those aspects of this embodied experience (Throsby, 2012a), and of shifting any anger or resentment about these procedures away from people who have the surgery and toward a system that views it as a “solution” (Kotow, 2024). This requires thinking about surgery’s ethics in a more entangled way; participants, in describing how the surgeries have helped and harmed them, affirm their right to access surgery albeit with better informed consent, awareness of potential lifelong surgical impacts, follow-up support, and most importantly, better advocacy, thus opening space for rapprochement between obesity science and fat studies.

Ultimately, our research shows that asking decontextualizing questions such as whether bariatric surgery is “right” or “wrong,” or whether patients are satisfied or dissatisfied with it might be a mistaken approach. Although these questions are not inherently wrong, they should not come at the expense of determining how to make non-normative, including fat, life liveable in the here and now, not just in some uncertain non-fat future. We need to ask questions about the current unliveability of fatness, and about how we shift the built environment, social relations, and healthcare policies/practices to create more meaningful and respectful inclusion of fat bodies. How do we make fat life more liveable is a much harder question to answer than whether weight loss surgery is “right” or “wrong,” and will require both obesity science and fat studies to engage with the materiality, biology, and affect of both fatness and this surgical experience.

## Notes

1. We refer to people who have received surgery as patients, recipients, or clients in recognition that all such terminology has been problematized within disability and critical health studies (Evans, 2004).

2. We align with other fat studies scholars who reject the unproblematized use of the term “obesity,” which carries biomedical and shaming connotations, and use the term in quotations upon first mention to denote our rejection.

3. Intra-activity is a term used in neomaterialism which was coined by Karen Barad (2007) as an alternative to interaction to refer to the inherently entangled nature of things, people, and forces. These agencies do not exist on their own but emerge through their intra-action.
